# Molecular mechanism of circRNAs in drug resistance in renal cell carcinoma

**DOI:** 10.1186/s12935-022-02790-w

**Published:** 2022-11-24

**Authors:** Shuang Qin, Yuting Wang, Peijun Wang, Qi Lv

**Affiliations:** Department of Medical Imaging, Tongji Hospital, Tongji University School of Medicine, Xincun Road No. 389, Shanghai, 200065 China

**Keywords:** RCC, Drug resistance mechanism, CircRNA, Research progress

## Abstract

Renal cell carcinoma (RCC) is one of the most common malignant tumors with a poor response to radiotherapy and chemotherapy. The advent of molecular targeted drugs has initiated great breakthroughs in the treatment of RCC. However, drug resistance to targeted drugs has become an urgent problem. Various studies across the decades have confirmed the involvement of circular RNAs (circRNAs) in multiple pathophysiological processes and its abnormal expression in many malignant tumors. This review speculated that circRNAs can provide a new solution to drug resistance in RCC and perhaps be used as essential markers for the early diagnosis and prognosis of RCC. Through the analysis and discussion of relevant recent research, this review explored the relationship of circRNAs to and their regulatory mechanisms in drug resistance in RCC. The results indicate an association between the expression of circRNAs and the development of RCC, as well as the involvement of circRNAs in drug resistance in RCC.

## Background

Renal cell carcinoma (RCC) is one of the most common malignant tumors [1, 2] that respond poorly to radiotherapy and chemotherapy effect [3]. Currently, tyrosine kinase inhibitor (TKI) drugs, such as sunitinib and sorafenib, are the most commonly used molecular targeted drugs to treat RCC. However, with the massive application of targeted drugs in clinical practice, drug resistance has gradually become an important concern in targeted drug use. Over the years, many researchers have explored the mechanisms of targeted drug resistance in RCC at the cellular and molecular levels. Studies have shown that the development of various tumors, such as glioblastoma, hepatocellular carcinoma, lung carcinoma, and breast carcinoma, is closely related to the expression of circular RNAs (circRNAs) [4–7]. This review explores the relationship between the molecular mechanism of circRNAs and the mechanism of drug resistance to targeted drugs in RCC and describes the association of circRNAs with the occurrence of drug resistance in RCC.

### Biological characteristics of circRNAs

CircRNAS are formed by the reverse shearing of precursor RNAs from end to end, which creates a very stable ring structure [8], 9]. CircRNAs are spatio-temporal and are variously expressed in different tissues and cells [10], 11]. CircRNAs can be divided into EcRNAs, CiRNAs, and EIcRNAs. CircRNAs are involved in cellular function, mainly through competitive endogenous RNA mechanisms [12–14]. CircRNAs act as the “sponges” of miRNAs because circRNAs have abundant miRNA loci on the circRNA ring structure, which can bind with various miRNAs, thereby influencing the regulation of downstream target genes. In addition, circRNAs can also alter splicing patterns or mRNA stability by binding RNA-binding proteins (RBPs) related to mRNA regulation [15]. CircRNAs can also interact with RNA polymerase and regulate transcription [16, 17]. Although circRNAs are noncoding RNAs, some circRNAs can encode regulatory peptides [18], 19]. CircRNAs play an essential role in regulating tumor genesis, proliferation, invasion, metastasis, drug resistance, and prognosis [20–22].

### Association between circRNA expression and RCC

Since the discovery of circRNAs, many studies have shown their close association with the biological behavior of tumors. The relationship between circRNAs and RCC has also been confirmed by several studies involving RNA sequencing, which has revealed abnormal circRNA expression in many tumor tissues [23]. CircRNAs are also involved in the growth, reproduction, invasion, and death of tumor cells [24, 25]. The analysis of the abnormal expression of circRNAs in RCC, along with tumor stage, histological grade, metastasis, and prognosis, has shown that the level of abnormal circRNA expression is correlated with tumor development, proliferation, invasion, and apoptosis.

Studies have also demonstrated that the expression of circRNAs, such as circ PUM1, circRNA ZNF609, circPTCH1, circPCNXL2, circRNA_001287, and circRNA SCARB1, significantly changes in RCC (Table 1). Furthermore, the enhanced expression of these circRNAs can promote the development of RCC [25–30]. Using data from existing miRNA databases, researchers screened out circRNAs differentially expressed in tumor and normal cells and subsequently compared and analyzed the effects of differentially expressed circRNAs on RCC at the molecular, cellular, individual, and population levels. Li et al. [31] found that circTLK1 was overexpressed in RCC and that such overexpression was related to the clinical manifestations of and poor prognosis in malignant tumor progression. Their experiments revealed that circTLK1 functioned as a sponge for miR-136-5p and positively regulated CBX4 expression. The overexpression of miR-136-5p significantly inhibited the mRNA and protein expression of CBX4. Conversely, in RCC tissues, miR-136-5p was significantly downregulated, whereas CBX4 was upregulated. The contrasting expressions of miR-136-5p and CBX4 were positively correlated with tumor size, distant metastasis, and poor prognosis. Li et al. further confirmed that circTLK1 knockdown inhibited the migration and invasion of RCC cells. CBX4 (also called polycomb 2) is a small ubiquitin-related modifier E3 ligase that facilitates the sumoylation of other proteins involved in tumorigenesis [32, 33] and increases vascular endothelial growth factor A expression and angiogenesis in hepatocellular carcinoma cells by promoting the sumoylation of HIF-1a [34]. In breast cancer [35], CBX4 promotes cell growth and metastasis in vitro and in vivo by regulating the miR-137/Notch1 signaling pathway. The CircRNAs cRAPGEF5 [31], hsa-circ-0072309 [37], circ-AKT3 [38], circUBAP2 [39], and circHIPK3 [40] are seldom expressed in RCC tissues. The variable expression of circRNA in normal and tumor tissues and cells suggests that circRNAs can be used as tumor biomarkers [41].

**Table 1:** Role of circRNAs in regulating renal tumor cells

circRNA	Target	Function	References
circPUM1	miR-340-5p/FABP7	Induced RCC’s progression	ZENG et al. [25]
circ‐ZNF609	miR‐138‐5p/FOXP4	Induced RCC’s progression	Xiong et al. [26]
circPTCH1	miR-485-5p/MMP14	Induced RCC’s progression	Liu et al. [27]
circPCNXL2	miR‐153/ZEB2	Induced RCC’s progression	Zhou et al. [28]
circRNA_001287	miR-144/CEP55	Induced RCC’s progression	Feng et al. [29]
circRNA SCARB1	miR- 510-5p/SDC3	Induced RCC’s progression	Sun et al. [30]
circTLK1	miR-136-5p	Induced RCC’s progression	Li et al. [31]
cRAPGEF5	miR-27a-3p/TXNIP	Suppressed RCC’s progression	Chen et al. [36]
hsa-circ-0072309	miR-100/PI3K/AKT and mTOR	Suppressed RCC’s progression	Tao et al. [37]
circ‐AKT3	miR‐296‐3p/E‐cadherin	Suppressed RCC’s progression	XUE et al. [38]
circUBAP2	miR-148a-3P/FOXK2	Suppressed RCC’s progression	SUN et al. [39]
circHIPK3	miR-637	Suppressed RCC’s progression	Li et al. [40]
hsa_circ_0035483	hsa-miR-335/CCNB1	Enhanced RCC’s resistance to gemcitabine	Yan et al. [57]
circSNX6	miR-1184/GPCPD1	Enhanced RCC’s resistance to sunitinib	Huang et al. [58]
circRNA-001895	miR-296-5P/FOX2	Enhanced RCC’s resistance to sunitinib	Tan et al. [59]
circEHD2	miR-4731-5p/ABCF2	Enhanced RCC’s resistance to sunitinib	Li et al. [61]
circME1	malic enzyme 1	Enhanced RCC’s resistance to sunitinib	Zhang et al. [63]

### CircRNAs and the mechanisms of drug resistance in RCC

Drug resistance in tumors is a common cause of therapeutic failure. During tumor drug therapy, possible mechanisms of drug resistance include the abnormal activation of tumor stem cells, increased metabolic rate of chemotherapeutic drugs, enhanced repair ability after DNA damage, loss of activity of apoptotic signaling pathways, redistribution of intracellular drug accumulation, and increased expression of transporters that recognize and exclude drugs [42–44]. Sanger et al. [45] first discovered circRNAs in the 1970s, whereas Hsu et al. [46] observed the circular structure of circRNAs in Hela cells under electron microscopy. Studies in recent decades have consistently confirmed the relationship between circRNAs and tumors. In addition to influencing the malignant progression of tumors, circRNAs have been associated with tumor drug resistance [47–50]. This suggests that circRNAs may function as regulatory agents of drug resistance in human cancers [51].

As competing endogenous RNAs (ceRNAs), circRNAs mostly perform their normal biological functions through circRNA–miRNA–mRNA regulation networks. miRNA are considered essential in a variety of biological processes in the body and important factors affecting the normal functioning of cells, including participation in the generation and progression of diseases [52–54]. CircRNAs are abundant in miRNA sites, which means that they can bind to various miRNAs with various roles in cells, promote or inhibit the expression of target genes, and thus regulate pathological processes in cells and the body [55, 56]. Studies that explored the relationship between the expression level of circRNAs and the clinical efficacy of RCC have found an association of many circRNAs with drug resistance in RCC and have described the mechanisms involved.

**Fig. 1:**
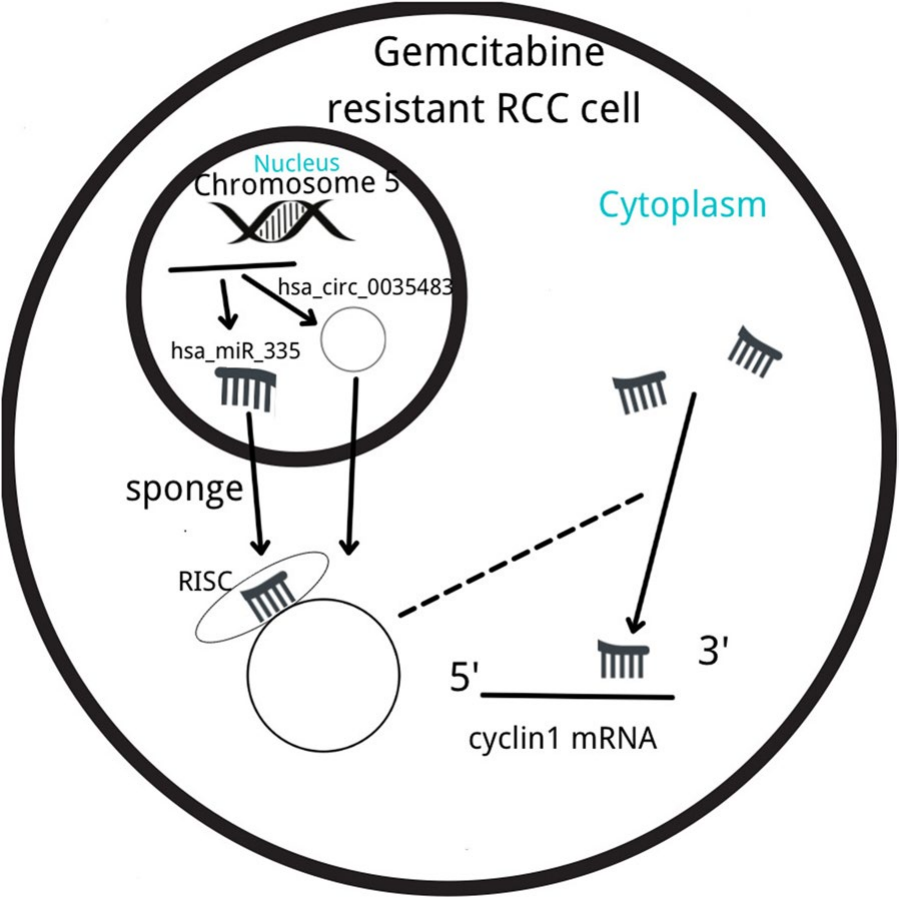
hsa_circ_0035483 and gemcitabine resistance in RCC

Yan et al. [57] analyzed circRNAs variously expressed in RCC by high-throughput sequencing and further investigated hsa_circ_0035483. The expressions of hsa_circ_0035483, hsa-miR-335, cyclin B1 (CCNB1), and the autophagy-related proteins were detected by RT-PCR or Western blot. Yan et al. further confirmed that hsa_circ_0035483 promoted autophagy by binding to hsa-miR-335 and enhanced gemcitabine resistance in RCC by promoting CCNB1 expression (Fig. 1). However, silencing hsa_circ_0035483 enhanced sensitivity to gemcitabine in vivo.

The circSNX6/miR-1184/GPCPD1 axis plays a crucial role in regulating intracellular LPA levels and sunitinib resistance in RCC. Specifically, Huang et al. [58] found that circSNX6 promoted sunitinib resistance in RCC by suppressing the inhibitory effect of miR-1184 on its target gene, GPCPD1, and increasing intracellular lysophosphatidic acid (LPA) levels. Tan et al. [59] showed that circRNA-001895 expression in sunitinib-resistant RCC was higher than that in chemotherapy-sensitive tissues. Upregulated circRNA-001895 expression in tumor cells was related to sunitinib resistance in RCC through controlled trials. Chen et al. [60] revealed that circRNA-001895 expression in tumor cells was related to sunitinib resistance in RCC and that hsa-circ-001895 regulated the downstream target gene *FOX2* through miR-296-5P. Li et al. [61] reported increased circEHD2 expression in sunitinib-resistant cell lines and tissues, which was linked to sunitinib resistance. Conversely, the knockdown of CircEHD2 reduced the progression of sunitinib-resistant cancer cells. Li et al. further reported that miR-4731-5p has a repressive function in RCC and reduces sunitinib resistance by targeting ABCF2, a member of the ABCF transporter family, which is a subgroup of the ATP-binding cassette transporter superfamily. ABCF2 is linked to drug resistance in several cancers [62]. The investigation further confirmed that ABCF2 was upregulated in RCC cells and that it mitigated the inhibitory effect of circEHD2 knockdown on sunitinib resistance in RCC. Additionally, they found that circEHD2 binds with miR-4731-5p in RCC, thereby confirming the essential role of circEHD2 in sunitinib resistance in RCC. However, Li et al.’s study could not establish a clear relationship between ABCF2 and circEHD2. CircRNAs indirectly regulate RCC by regulating the expression and activity of tumor-related target genes through a regulatory network mediated by miRNAs. These circRNAs maintain intracellular homeostasis in physiological states. Once their expression changes in RCC cells, they will not only promote tumor development, proliferation, invasion, and metastasis but also significantly increase the probability of drug resistance in tumor cells.

CircRNAs can regulate cellular physiological and pathological processes through ceRNA mechanisms and by binding to RBPs and RNA polymerase. Furthermore, ribosomes can translate some circRNAs and encode peptides to enable them to perform regulatory functions. Zhang et al. [63] identified a novel circRNA named circME1, which was highly expressed in sunitinib-resistant clear-cell RCC cells, and found that circME1 promotes aerobic glycolysis and sunitinib resistance in clear-cell RCC through the cis-regulation of malic enzyme 1 (ME1). CircME1 enhanced the expression of its parental gene ME1 in cis-regulation by interacting with U1 snRNP at the promoter of ME1. Aerobic glycolysis, also known as the Warburg effect, is involved in tumor progression and the development of sunitinib resistance [64–66]. The role of noncoding RNA-mediated overexpression of ABC transporters in chemotherapy-resistant tumors also cannot be ignored [67].

A potential mechanism of chemoresistance or targeted drug resistance in tumor cells may be the cytoprotective functions of autophagy. Furthermore, circRNAs are variously expressed in response to cisplatin, suggesting their involvement in the pathophysiology of cisplatin-induced nephrotoxicity [68]. A study investigated the potential impact of radiation therapy on circRNA expression and reported that the irradiation of human embryonic kidney cells resulted in a clear variation in circRNA expression signatures [69]. These data suggest a possible involvement of circRNAs in treatment resistance in RCC, but further studies are needed to clarify these relationships.

The tumor angiogenesis theory proposed by Folkman in the twentieth century pointed out that tumor angiogenesis is an essential process of tumor growth [70, 71]. Currently, the most widely used TKI drugs in the treatment of RCC have been developed based on this principle. A VEGF/VEGFR-targeted antibody specifically binds VEGF/VEGFR to inhibit the downstream signaling pathway, thus inhibiting the generation of tumor blood vessels and limiting tumor growth. However, with the massive application of targeted drugs in clinical practice, drug resistance has gradually become an important concern in targeted drug use. Few literature reviews have described the role of circRNAs in the mechanism of sunitinib resistance in RCC. This may be a valuable line of further research.

## Conclusions

The development of molecular targeted drugs has dramatically improved the therapeutic outcomes for RCC. Still, increasing resistance to targeted drugs has become an urgent problem. Drug resistance is a complex process, and the activation of multiple pro-angiogenic pathways may be associated with TKI resistance. This review discussed the role of circRNAs in the mechanism of drug resistance in RCC. CircRNAs were once thought to be the product of RNA mishearing [72]. However, several studies have shown that circRNA plays a vital role in both physiological and pathological states and is associated with tumorigenesis in various cancers, including RCC.

In conclusion, current studies suggest that circRNAs play a crucial role in mediating drug resistance in RCC. CircRNAs enhance RCC’s resistance to sunitinib and gemcitabine primarily through ceRNA mechanisms. Further studies are required to elucidate further the involvement and mechanisms of circRNAs in drug resistance in RCC. The research and discussion on the involvement of circRNAs in the mechanism of anti-tumor drug resistance provides new insights on developing strategies to overcome drug resistance in clinical practice, as well as on developing more effective treatments for RCC.

## Data Availability

Not applicable.

## Abbreviations

RCC: Renal cell carcinoma
TKI: Tyrosine kinase inhibitors
VEGF: vascular endothelial growth factor
VEGFR: vascular endothelial growth factor receptor
RBPs: RNA binding protein
ceRNAs: Competing endogenous RNAs
CCNB1: Cyclin B1
GPCPD1: Glycerophosphocholine phosphodiesterase 1
LPA: Lysophosphatidic acid
